# Promoter methylation inhibits expression of tumor suppressor KIBRA in human clear cell renal cell carcinoma

**DOI:** 10.1186/s13148-017-0415-6

**Published:** 2017-10-06

**Authors:** Katrin Schelleckes, Boris Schmitz, Giuliano Ciarimboli, Malte Lenders, Hermann J. Pavenstädt, Edwin Herrmann, Stefan-Martin Brand, Eva Brand

**Affiliations:** 1 Internal Medicine D, Nephrology, Hypertension and Rheumatology, University Hospital Muenster, Albert-Schweitzer-Campus 1, 48149 Muenster, Germany; 2 Institute of Sports Medicine, Molecular Genetics of Cardiovascular Disease, University Hospital Muenster, Horstmarer Landweg 39, 48149 Muenster, Germany; 3 Clinic for Urology, University Hospital Muenster, Albert-Schweitzer-Campus 1, 48149 Muenster, Germany

**Keywords:** Epigenetics, Renal cancer, Hippo pathway, Methylation, Tumor, WWC1

## Abstract

**Background:**

KIBRA has been suggested as a key regulator of the Hippo signaling pathway, regulating organ size, cell contact inhibition, tissue regeneration as well as tumorigenesis and cystogenesis. We recently reported that human *KIBRA* expression depends on a complex alternative CpG-rich promoter system. Our current study aimed at the identification of epigenetic mechanisms associated with alterations in *KIBRA* expression regulation.

**Results:**

We identified two separated methylation-sensitive CpG islands located to independent *KIBRA* promoter regions. In vitro promoter methylation analysis using human neuroblastoma (SH-SY5Y) and immortalized kidney cells (IHKE) revealed that total promoter methylation by CpG methyltransferase *Sss*I resulted in complete abrogation of transcriptional activity (*p* < 0.001), while partial methylation by *Hpa*II selectively repressed *KIBRA* core promoter activity in kidney cells (*p* < 0.001). Cell culture-based experiments demonstrated that 5-azacitidine may be used to restore KIBRA mRNA and protein levels, while overexpression of transcription factor SP1 also induced *KIBRA* upregulation (all *p* < 0.001). Furthermore, SP1 transactivation of *KIBRA* transcription was largely prevented by methylation of *KIBRA* regulatory elements (*p* < 0.001). Analysis of human kidney biopsies revealed that *KIBRA* promoter methylation was associated with human clear cell renal cell carcinoma (ccRCC; *n* = 8 vs 16 controls, OR = 1.921, [CI 95% = 1.369–2.695]). The subsequent determination of KIBRA mRNA levels by real-time PCR in a larger patient sample confirmed significantly reduced KIBRA expression in ccRCC (*n* = 32) compared to non-neoplastic human kidney tissue samples (controls, *n* = 32, *p* < 0.001).

**Conclusion:**

We conclude that epigenetic downregulation of tumor suppressor KIBRA may involve impaired SP1 binding to functional methylation-sensitive *KIBRA* promoter elements as observed in human kidney clear cell carcinoma. Our findings provide a pathophysiological basis for future studies on altered *KIBRA* regulation in clinical disease entities such as renal cancer.

**Electronic supplementary material:**

The online version of this article (10.1186/s13148-017-0415-6) contains supplementary material, which is available to authorized users.

## Background

KIBRA (WWC1), a WW and C2 domain-containing protein has been identified as an upstream regulatory component of the Hippo pathway (also known as Salvador-Warts-Hippo tumor suppressor network), which regulates cell number by modulating proliferation, apoptosis, and differentiation [1–4]. Initially, the Hippo pathway has been defined in *Drosophila melanogaster* by tumor suppressor gene screenings. The inactivation of Hippo genes including *Warts* (*Wts*), *Hippo* (*Hpo*), *Salvador* (*Sav*), and *Mats* as well as *Merlin* (*Mer*) and *Expanded* (*Ex*) resulted in a comparable phenotype with considerable tissue overgrowth [5]. The Hippo pathway is highly conserved in mammals, and the ability of the WWC proteins to modulate Hippo signal transduction and thus to inhibit cell proliferation has been proposed to be evolutionarily conserved from fly to men [5, 6]. The Hippo pathway negatively regulates the activity of two main downstream mediators: Yes-associated protein (YAP) and its family member the transcriptional co-activator with PDZ-binding motif (WWTR1/TAZ) [7–9]. Upon phosphorylation, YAP and TAZ promote proliferation and inhibit apoptosis by interaction with different transcription factors, including TEA domain family member (TEAD) 1–4 [10]. KIBRA acts as an upstream tumor suppressor protein that regulates Hippo signaling in conjunction with neurofibromatosis-2 (NF2), potentially preventing YAP and TAZ activation [1–3].

In humans, impaired Hippo signaling has been reported in a variety of different cancers, linking deregulated Hippo signaling to tumor initiation and progression [11–16]. Components of the Hippo pathway have, therefore, been suggested to be the target of aberrant gene methylation and epigenetic silencing in humans [13] as already reported for *LATS1/2* (large tumor suppressor kinases 1 and 2; human *Warts* homolog) [17, 18], *MST1/2* (serin/threonine protein kinase 4/3; human Hippo homolog) [19], and *KIBRA* [20, 21].

Since DNA methylation, especially at promoter-associated CpG islands of tumor suppressors has been recognized as a major contributor to cancer development, we analyzed the consequences of human *KIBRA* promoter methylation and determined the methylation status of *KIBRA* promoters in patients with clear cell renal cell carcinoma (ccRCC).

## Results

### Identification of CpG islands within functional KIBRA promoter regions

**Fig. 1:**
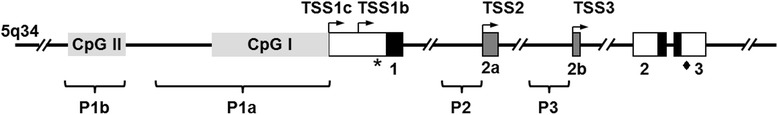
CpG islands within *KIBRA* promoter P1a and P1b. The alternative *KIBRA* promoter system composed of promoter regions P1a, P1b, P2, and P3 and their respective transcription start sites (TSS; right-angled arrows; filled boxes represent exons) located on chromosome 5. Two CpG islands with a length of 205 bp (CpG II, position − 3594/− 3799) and 764 bp (CpG I, position − 1/− 764) within the *KIBRA* promoter regions P1a and P1b were identified. Of note, Figure S1 contains detailed description of CG dinucleotide rate and positions within CpG islands

The current analysis of CpG residues was based on our characterization of the complex structure of the human *KIBRA* promoter system [22]. This previous analysis revealed that renal *KIBRA* gene expression is driven from a constitutively active core promoter (P1a) and at least one alternative promoter (P1b). Notably, the isolated alternative promoter P1b was cell type-specific and appeared to be silenced in neuronal cell lines. In the current analysis, an in silico screen of these active promoters revealed two CpG islands with a length of 764 bp (CpG I) and 205 bp (CpG II) within the *KIBRA* promoter regions P1a and P1b, respectively (Fig. 1; Additional file 1: Figure S1).

### KIBRA CpG I methylation and expression is associated with ccRCC

**Fig. 2:**
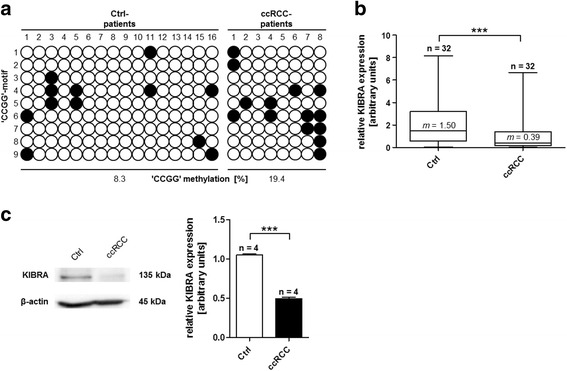
*KIBRA* “CCGG” methylation within CpG I in ccRCC samples. **a** Methylation patterns of “CCGG”-motifs within CpG I in control (Ctrl) and ccRCC samples. Each line represents one “CCGG”-motif within CpG I. Filled circles indicate methylated, open circles indicate unmethylated “CCGG”-motifs. Differences of methylation between the control (12/144 [8.3%]) and ccRCC (14/72 [19.4%]) group were significant (*p* = 0.039). Analysis revealed significantly decreased mRNA KIBRA expression levels for ccRCC samples in **b** real-time PCR and **c** western blot analyses. ***p < 0.001; *m* median

Since ccRCC is the most common histological subtype of adult kidney cancer and promoter regions of several tumor suppressor genes have already been reported to be frequently methylated in primary ccRCC tumor samples [23–25], we determined specific *KIBRA* methylation patterns in human ccRCC. The methylation analysis in human ccRCC samples was focused on CpG I, which harbors the constitutively active core promoter (P1a) of *KIBRA*. To characterize the methylation state of CpG I (63 CG dinucleotides within P1a), we evaluated methylation patterns in 16 non-neoplastic human kidney tissue samples (adjacent benign regions, control) and eight human ccRCC samples in detail by bisulfite sequencing. ccRCC cells are arranged in compact nests, sheets, alveolar, or acinar structures and have clear cytoplasm which distinguishes them microscopically from adjacent benign regions (Additional file 1: Figure S2) [26]. However, subcellular changes including gene methylation might also be observed in phenotypically unchanged regions. Bisulfite-converted DNA of each sample was subcloned and plasmid DNA of five colonies per patient were sequenced. Using this sensitive approach, cellular differences in the methylation state of each patient’s tissue sample could be detected. Bisulfite sequencing and dichotomous analysis revealed that *KIBRA* CpG methylation occurred significantly more often in ccRCC samples (methylation level 73/504 [14.5%]) compared to control samples (methylation level 76/1008 [7.5%]; *p* = 0.0001, OR = 1.921, [CI 95%] = 1.369–2.695). Further analysis of “CCGG” methylation within CpG I revealed that methylation also occurred significantly more often at this specific motif in ccRCC samples (methylation level 14/72 [19.4%]) compared to controls (methylation level 12/144 [8.3%]; *p* = 0.0390, OR = 2.333, [CI 95%] = 1.026–5.306; Fig. 2a). Additional parametric analysis of CpG and “CCGG” methylation confirmed these results (data not shown).

A subsequent analysis of KIBRA tissue mRNA levels by real-time PCR analysis revealed significantly reduced *KIBRA* expression levels in ccRCC (*n* = 32) compared to non-neoplastic human kidney tissue samples (*n* = 32, *p* < 0.001, ES 0.7; Fig. 2b). Post hoc power calculation revealed a power of 1-beta = 0.8 for the analyzed 64 samples (alpha = 0.05, two-sided *t* test). An additional analysis on protein level supported these findings in that KIBRA protein was significantly decreased in ccRCC samples (*n* = 4) compared to adjacent benign tissue samples (*n* = 4, *p* < 0.001; Fig. 2c).

### CpG methylation leads to transcriptional KIBRA inhibition

**Fig. 3:**
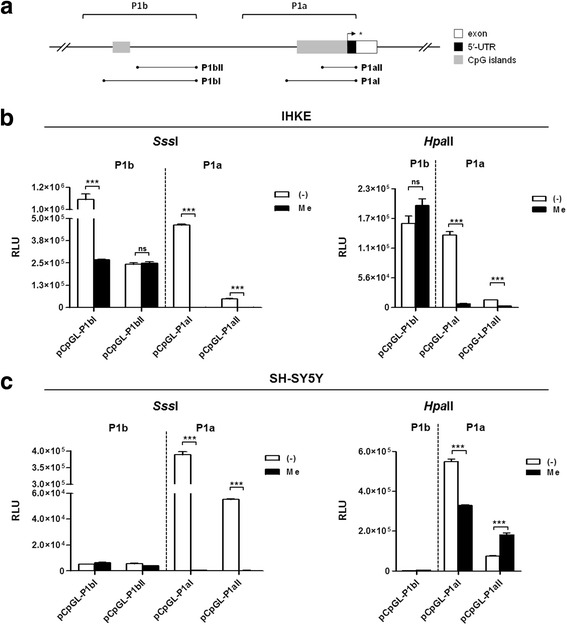
Transcriptional inhibition of *KIBRA* promoters by in vitro methylation. **a** Schematic representation of *KIBRA* promoters P1a and P1b, CpG islands, and respective positions of methylated promoter constructs generated in the CpG-free pCpGL-Basic vector. **b** In renal IHKE cells, transcriptional activity of promoter P1a was extensively silenced by *Sss*I methylation. No considerable differences were observed for transcriptional activity of control region P1bII (without CpG island). Transcriptional activity of promoter portion P1bI was significantly decreased. Partial methylation by *Hpa*II also resulted in a significant decrease of P1a transcriptional activity, whereas P1bI was unaffected. **c** Transcriptional activity of P1a was comparably silenced by *Sss*I methylation in neuronal SH-SY5Y cells. Partial methylation by *Hpa*II resulted in an increase of transcriptional activity of promoter region P1aII, whereas transcriptional activity of P1aI was significantly decreased. None of the P1b promoter constructs exerted sufficient activity in SH-SY5Y cells, independent of the methylation pattern. Figures are representative for experiments (*n* = 3). Transcriptional activity was assessed as relative light units (RLU). ***p < 0.001, *ns* not significant

To investigate the consequences of CpG methylation on *KIBRA* expression, we inserted active *KIBRA* promoter regions into the pCpGL-Basic reporter gene vector [27]. The pCpGL-Basic vector was chosen instead of the conventional pGL3-Basic vector due to a CpG dinucleotide-free backbone, which allows observation of methylation effects on the inserted promoter fragments rather than detecting artificial effects of reporter gene (i.e., *luciferase*) methylation. For each promoter region, two reporter gene vectors were generated (Fig. 3a): P1aI covering the complete CpG I and P1aII covering the proximal part of CpG I. P1bI harbored the complete CpG II, while P1bII did not include any CpG island and served as negative control for methylation effects. In vitro methylation of CpG I by methyltransferase *Sss*I (methylation of all CG-dinucleotides) led to total abrogation of P1aI and P1aII transcriptional activity compared to the unmethylated promoter in renal IHKE cells (Fig. 3b; *p* < 0.001). The effect was also observed in neuronal SH-SY5Y cells (Fig. 3c; *p* < 0.001). Target specific methylation of “CCGG”-motifs using *Hpa*II led to a similar inhibition of promoter activity in IHKE cells (Fig. 3b; *p* < 0.001), while residual activity for P1aI was observed in SH-SY5Y cells (Fig. 3c; *p* < 0.001). Methylation of distal CpG II also led to a significant reduction of P1bI transcriptional activity compared to the active unmethylated promoter (*p* < 0.001). P1bII, which served as negative methylation control, was not affected by methylation. No difference in transcriptional activity was observed after *Hpa*II methylation of CpG II (Fig. 3b).

### KIBRA expression is induced by DNA methyltransferase inhibitor 5-azacitidine

**Fig. 4:**
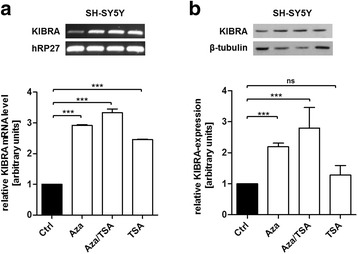
*KIBRA* expression is activated by demethylation using 5-azacytidine. **a** DNA demethylation by 5-azacytidine (Aza) and inhibition of histone deacetylases by trichostatin A (TSA) resulted in ~3-fold increase in KIBRA mRNA levels in neuronal SH-SY5Y cells. Cells were treated with 5 μM Aza for 3 days and 250 nM TSA for the last 24 h. *KIBRA* expression levels were analyzed by PCR and subsequent densitometry compared to hRP27 control. **b** Effect of DNA demethylation by Aza and inhibition of histone deacetylases by TSA on KIBRA protein expression detected by western blot analysis. KIBRA protein levels increased significantly after treatment with Aza and Aza/TSA. Figures are representative for experiments (*n* ≥ 3); western blot densitometry is based on three individual experiments. **p < 0.01, ***p < 0.001, *ns* not significant

Since *KIBRA* promoter regions are partly silenced in SH-SY5Y cells [22], we used SH-SY5Y neuroblastoma cells as a model cell line to analyze the effects of whole genome demethylation on *KIBRA* expression. Treatment of SH-SY5Y cells with 5-azacytidine (Aza), alone or in combination with trichostatin A (TSA), an inhibitor of histone deacetylases, resulted in a significant ~3-fold increase in KIBRA mRNA levels compared to untreated cells (Fig. 4a; *p* < 0.001). This observation was confirmed for the Aza/TSA application on protein level by western blot (Fig. 4b; all *p* < 0.01 compared to control), suggesting that promoter demethylation restores *KIBRA* expression. Of note, TSA alone changed the KIBRA mRNA but not the protein expression level.

### SP1 transactivation is prevented by KIBRA promoter methylation

**Fig. 5:**
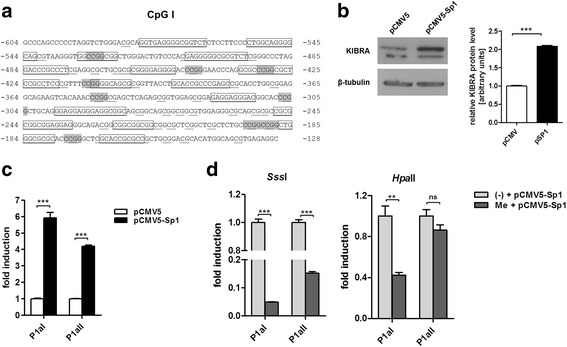
SP1 transactivation is prevented by in vitro methylation. **a** In silico analysis revealed conserved SP1 binding sites (boxed) within CpG I. CpG dinucleotides are underlined and “CCGG”-motifs are marked in gray. **b** SP1 overexpression resulted in elevated KIBRA protein levels detected by western blot. **c** Transcription of *KIBRA* is stimulated by SP1. SP1 overexpression in IHKE cells resulted in significant transcriptional activation of P1aI, and P1aII promoter constructs harboring CpG I compared to pCMV5 mock-control. **d** In vitro methylation of promoter fragments by *Sss*I prevented the stimulatory SP1 effect significantly, while partial methylation by *Hpa*II selectively affected P1aI. Figures are representative for experiments (*n* = 3); western blot densitometry is based on three individual experiments. **p < 0.01, ***p < 0.001, *ns* not significant

Based on the identified CpG islands in the functional *KIBRA* promoters, we conducted an in silico analysis for SP1 binding sites in these regions. Several conserved SP1 binding sites were detected in CpG I, while CpG II revealed only three potential SP1 binding sites (mean conservation level ≥ 80%; Fig. 5a). Subsequent overexpression of SP1 in IHKE cells resulted in strong activation of promoter P1a and P1b (Fig. 5c; *p* < 0.001 compared to control). This effect was also confirmed by western blot analysis as KIBRA protein level was increased after SP1 overexpression (Fig. 5b; *p* < 0.001). Promoter methylation by *Sss*I and also *Hpa*II significantly prevented SP1 transactivation (Fig. 5d; *p* < 0.001). This observation suggests that SP1 recruitment to *KIBRA* promoter regions is prevented by CpG methylation.

## Discussion

Recent data underlined the critical role of KIBRA as an upstream regulator of the Hippo signaling pathway. Therefore, decrease or loss of *KIBRA* expression and/or function may have a considerable impact on tumorigenesis or tumor progression [28–30]. The goal of this study was to elucidate epigenetic effects on *KIBRA* expression regulation in vitro and in human ccRCC.

The correlation between promoter methylation and gene silencing has already been extensively demonstrated [31, 32]. The molecular mechanisms of this methylation-induced gene inactivation involve histone modification by histone deacetylases and non-covalent linkage between positively charged histones and negatively charged DNA. As a result, transcription factors are hindered in accessing their binding sites leading to reduced or silenced gene expression [33]. This mechanism may be reverted by TSA, an inhibitor of histone deacetylases, when used with the demethylating agent Aza, as we were able to show for KIBRA using SH-SY5Y neuroblastoma cells as a model cell line. In our experiments, TSA treatment alone activated KIBRA mRNA expression in vitro after a single dose of 250 mmol/l for 24 h. The effect was not observed on protein level with TSA alone but KIBRA protein was elevated using the combination of Aza/TSA. Interestingly, a comparable effect has been described in vivo for survival motor neuron protein (smn) gene expression in mice in that a single dose of TSA only affected smn RNA levels while repeated treatment of these mice with TSA stabilized elevated smn protein levels [34]. This effect might also be explained by the observation that SP1 is activated by TSA in a dose-dependent manner as described for the human ELK1 promoter [35]. In this respect, methylation at the CG dinucleotide of a specific transcription factor binding element such as SP1 consensus sites may sterically interfere with binding of the transcription factor to DNA, inhibiting transcription [36]. With regard to *KIBRA* methylation, the group of Latif and colleagues recently identified frequent epigenetic inactivation of *KIBRA* in B cell acute lymphocytic leukemia and unfavorable prognostic parameters in chronic lymphocytic leukemia [20, 21]. In their methylation analysis, the authors addressed a 368 bp region of *KIBRA* based on TSS1a (NM_015238), a region mapping to promoter P1a.

**Fig. 6:**
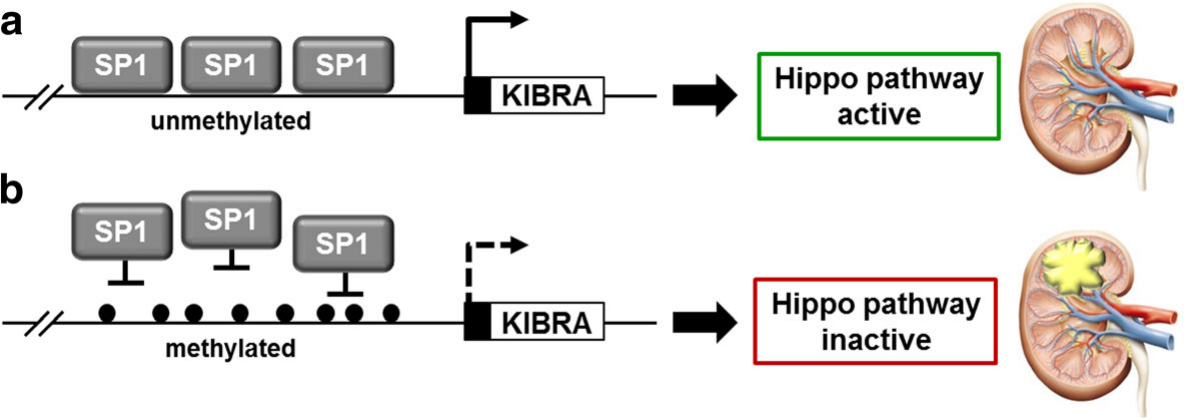
A proposed model for Hippo pathway regulation by *KIBRA* promoter methylation. **a** SP1 binds to conserved DNA recognition elements within *KIBRA* promoter regions leading to enhanced *KIBRA* expression and Hippo pathway activation. **b** SP1 recruitment to *KIBRA* promoter regions may be prevented by CpG methylation leading to reduced KIBRA expression and impaired Hippo pathway activation. Alterations of the Hippo pathway due to *KIBRA* promoter methylation may be associated with onset or progression of renal cell carcinoma

Our in vitro methylation experiments revealed that *KIBRA* expression is significantly affected by methylation patterns of at least two CpG islands located within functional promoters P1a and P1b. Transcription factor analysis revealed that SP1 is a strong activator of *KIBRA* promoter activity, and KIBRA protein levels were significantly elevated by SP1 overexpression. Upon methylation of P1a promoter elements, the activating effect of SP1was lost. Moreover, “CCGG” methylation was sufficient to prevent transcriptional stimulation by SP1. Our results, therefore, suggest that in vivo methylation at *KIBRA* CG dinucleotides of SP1 consensus sites may reduce the ability of SP1 to bind its DNA recognition element potentially impairing transactivation (Fig. 6). The role of the SP transcription factor family in different cancers has already been highlighted [37], and there is evidence that overactivation of SP1 occurs frequently in a wide variety of different tumors, correlating with aggressive biology and poor clinical outcome of these tumors [38–40]. In ccRCC, a potential tumor suppressor function of KIBRA might be absent even in the presence of high SP1 expression levels if *KIBRA* promoter regions are deactivated by methylation.

Alterations of *KIBRA* expression in ccRCC has already been analyzed in whole-genome expression profiling using Illumina BeadChip technology. The gene expression profiles of 101 ccRCC and adjacent tissue sample pairs of the K2 series suggested KIBRA downregulation in this series using locus-specific probes [41]. However, the detected signal has not been validated using real-time PCR. Our analysis confirms KIBRA downregulation in ccRCC and provides evidence for an association of reduced KIBRA mRNA levels with the significantly increased *KIBRA* promoter methylation. Of note, significantly increased *KIBRA* methylation in ccRCC was additionally confirmed by calculation of individual methylation grade (data not shown). Our findings provide a potential basis for future clinical studies which should analyze if *KIBRA* methylation or expression can be associated with ccRCC survival rates, tumor stage, or size. Since ccRCC is often fatal once metastatic [42], it is important to detect ccRCC at early stages when lesions are small. In this respect, other studies suggested the implementation of a “DNA methylation biomarker panel” [29, 43]. If aberrant *KIBRA* methylation or reduced *KIBRA* expression can also be detected in blood or cells isolated from urine of ccRCC patients is currently unclear and further studies are needed to investigate these potential correlations. If such a correlation exists, alterations in *KIBRA* methylation or expression patterns might also be useful for differential diagnosis of other RCC entities.

### Limitations

ccRCC tissues exhibit substantial heterogeneity regarding both histological (fractions of different tissue) and molecular aspects (different methylation pattern of alleles). This aspect might be associated with the diverse KIBRA mRNA expression levels in the analyzed control samples. This limitation may be addressed by microdissection or cell sorting in subsequent analyses. Furthermore, obtained data may be limited to the analyzed Caucasian study cohort and relatively small sample sizes.

## Conclusion

We conclude that promoter methylation is a major mechanism involved in *KIBRA* expression regulation. *KIBRA* expression levels are reduced in ccRCC and alterations in the balance of *KIBRA*/SP1 binding by promoter methylation may be involved in the onset and/or progression of ccRCC. Based on these initial data, the scope of our future studies will be the expansion of ccRCC tissue sample numbers and patient data to correlate the detected methylation levels with patient’s clinical outcome. Furthermore, *KIBRA* expression and methylation levels will be analyzed in cultured ccRCC cells including Aza/TSA treatment for the reactivation of *KIBRA* expression.

## Methods

### Sample collection and preparation

Human ccRCC samples (female = 40.6%, mean age = 63 years) and adjacent benign normal kidney tissue samples (female = 43.8%, mean age = 66 years) were collected shortly after partial or radical nephrectomy at the outpatient surgery center (Clinic for Urology, University Hospital Muenster). Of note, ccRCC and control samples were obtained from different patients. All patients gave written informed consent, and the study was approved by the ethics committee of the Medical Faculty of the Westphalian Wilhelms-University of Münster, Germany (2008–030-f-S). All specimens were classified according to the UICC (Union Internationale Contre le Cancer) TNM staging system [44] and subclassified after histopathological work up and hematoxylin and eosin staining by a pathologist (Additional file 1: Table S1) [45]. Adjacent benign normal samples were harvested distant from the tumor and confirmed by histology to be free of contamination with malignant cells. Tumor samples were confirmed to be enriched (> 80% epithelial cells) for cancer cells relative to stroma. Tissue samples were snap-frozen in liquid nitrogen immediately and stored at − 80 °C.

### Tissue methylation analysis

The methylation status of *KIBRA* promoter regions in human renal biopsies was analyzed by bisulfite sequencing. Genomic DNA was prepared from 25 mg kidney tissue using the Qiamp DNA Blood Mini kit (Qiagen). Bisulfite conversion of 500 ng DNA was performed using the EpiTect Bisulfite Kit (Qiagen) according to the manufacturer’s protocol. PCR products were generated by KAPA HiFi Uracil+ DNA polymerase (PEQLAB) and CpG island-specific oligonucleotides (Additional file 1: Table S2). For sequencing, PCR products were ligated into the pGEM-T Easy vector (Promega) and plasmid DNA of five colonies per patient was isolated and sequenced using M13 oligonucleotides. Methylation data for CpGs or “CCGG”-motifs were treated as dichotomous or calculated parametrically as individual methylation grade. A position was defined as methylated for dichotomization, when at least one out of five CpGs or “CCGG”-motifs was methylation positive after bisulfite conversion and sequencing.

### Cell culture

IHKE cells were maintained in DMEM/Ham’s-F12 (Life Technologies) enriched with 5% fetal bovine serum (FBS; Sigma-Aldrich), 100 units/ml penicillin, 100 ng/ml streptomycin, 2 mmol/ml L-glutamine, 10 ml/l insulin­transferrin-sodium selenite media supplement, 1.25 g/l NaHCO_3_, 55 mg/l sodium pyruvate, 10 μg/l human epidermal growth factor (all Sigma-Aldrich), and 15 mmol/l *N*­2­hydroxyethylpiperazine­*N*­2­ethanesulfonic acid (HEPES; Merck) [46–48]. SH-SY5Y cells were maintained in DMEM with 20% FBS, 100 units/ml penicillin, 100 ng/ml streptomycin, and 2 mmol/ml L­glutamine. Genome demethylation was achieved using 5 μmol/l 5-azacitidine (Aza; Sigma-Aldrich) over 3 days with daily media changes. Trichostatin A (TSA, 250 nmol/l; Sigma-Aldrich) was applied for 24 h.

### In vitro methylation analysis

Deletion constructs of the *KIBRA* 5′-flanking region have been described elsewhere [22]. For methylation analyses, *KIBRA* promoter fragments were introduced in 5′-3′-orientation into the promoter-less, CpG dinucleotide-free luciferase reporter gene vector pCpGL-Basic [27]. Amplification of functional promoter fragments harboring the identified CpG islands was based on annotated TSS1a (NM_015238). In vitro methylated DNA was generated by incubating 10 μg of vector DNA with 12 units of *Sss*I or *Hpa*II methylase and 640 μM *S*-adenosyl-l-methionine (all New England Biolabs) at 37 °C for 6 h. After phenol-chloroform extraction, DNA pellets were resuspended in H_2_O to a final concentration of 1 μg/μl. IHKE and SH-SY5Y cells were transfected using JetPEI (PEQLAB) and 1 μg of methylated plasmid DNA per 24 well. The pCpGL-CMV vector [27] was used as control for transfection efficiency, the CpG island-free vector P1bII served as negative control. Luciferase activities were determined using a luciferase assay kit (Promega) and a Sirius luminometer (Berthold detection systems). For SP1 overexpression, pCMV5-SP1 or shuttle vector control pCMV5 and methylated reporter gene plasmids were transfected in a 1:1 ratio as described previously [49]. All vectors were sequenced to ensure sequence accuracy and identity. Transfection experiments were repeated at least three times.

### Western blot

For crude protein extracts, cells or tissue were lysed in RIPA buffer containing 1% NP40 and 0.1% sodium dodecyl sulfate (SDS) supplemented with “complete” protease inhibitor cocktail (Roche). Immunodetection of cellular extracts was performed using an anti-KIBRA (Santa Cruz Biotechnology; 1:500), anti-SP1 (Millipore; 1:1000), and anti-rabbit secondary antibody (GE Healthcare; 1:5000). Sample loading was confirmed by β-tubulin detection (cell signaling; 1:5000) and anti-mouse secondary antibody (GE Healthcare; 1:20,000). For the analysis of KIBRA protein levels in randomly selected patients’ tissue samples (*n* = 4), tumor or control tissue was homogenized (BANDELIN Sonoplus HD2070, BANDELIN electronic GmbH & Co. KG), pooled, and quantified by SDS page using anti-KIBRA antibody (1:250). Sample loading was confirmed by β-actin detection (cell signaling; 1:5000) and anti-mouse secondary antibody (GE Healthcare; 1:20,000). Western blots were repeated at least three times, and band intensities were assessed using Image J.

### Expression analysis

Total RNA was extracted from cells or tissue samples using the NucleoSpin RNA Kit (Macherey-Nagel). First strand cDNA synthesis was performed using Superscript II reverse transcriptase (Life Technologies) and 1 μg of total RNA. cDNA was amplified in a 384-well format (standard real-time PCR conditions) in duplicates using Power SYBR Green (Applied Biosystem) on an Applied Biosystems 7500 Fast Real-Time PCR System. Relative quantification was calculated using the 2^−ΔΔCt^ method and S18 as endogenous control. The absence of non-specific amplification products was confirmed by agarose gel electrophoresis and generation of melting curves using the Applied Biosystems software. Oligonucleotides had an amplification efficiency of ≥ 90% (Additional file 1: Table S2). In case of *KIBRA* expression analysis following 5-azacitidine/trichostatin A treatment, standard PCR with hRP27 loading control was used.

### Identification of CpG islands and SP1 binding sites

The *KIBRA* promoter regions P1a and P1b were analyzed using the CpG Island Searcher version 10/29/04 (http://cpgislands.usc.edu/) [50] with the following settings. Selected lower limits %GC = 55%, CpG_obs_/CpG_exp_ = 0.65, length > 200 bp, and distance = 100 bp. Identified CpG islands were analyzed for SP1 consensus sites by computer-aided analyses using AliBaba2.1 [50, 51], a position weight matrix algorithm based on the TRANSFAC database of eukaryotic transcription factors (TRANSFAC 7). Results were validated using PROMO 3.0.2 [51, 52].

### Statistical analysis

The magnitude of change in KIBRA expression between control and ccRCC samples was expressed as standardized effect sizes (ES), calculated from means and SD (Cohen’s d). Power calculations were performed using G*Power 3.1.9.2 [52, 53]. Methylation data for CpGs or “CCGG”-motifs were treated as dichotomous (methylated/unmethylated, Fig. 2a). Comparison between ccRCC samples and controls was performed using two-sided Chi-square test or Mann-Whitney *U* test, where appropriate. *p* values were calculated by unpaired, two-tailed Student’s *t* test or one-way ANOVA, where appropriate. Data are given as mean ± SD. *p* values < 0.05 were considered significant.

## Additional file

Additional file 1: Table S1. Clinicopathological characteristics of the study cohort. Table S2 Sequences and positions of oligonucleotides used in this study. Figure S1 *KIBR*A CpG islands were detected using “*CpG Island searcher*.” Two CpG islands were detected: CpG II with 205 bp and CpG I with 764 bp. Parameter settings: %GC = 55%, CpG_obs_/CpG_exp_ = 0.65, lengths > 200 bp, distance = 100 bp. Position of *KIBRA* promoter regions P1b and P1a is indicated according to TSS1a (NM_015238). Figure S2 Hematoxylin and eosin staining from adjacent benign tissue (Ctrl; I/II) and ccRCC tissue (III/IV) used for the analysis (DOCX 3044 kb)

## Abbreviations

Aza: 5-azacytidine
ccRCC: Clear cell renal cell carcinoma
KIBRA: Kidney and brain
SP1: Specificity protein 1
TSA: Trichostatin A
TSS: Transcription start sites.
