# Supplementary material for: Promoter methylation inhibits expression of tumor suppressor KIBRA in human clear cell renal cell carcinoma
Source: Clin Epigenetics. 2017 Oct 6;9:109. doi: 10.1186/s13148-017-0415-6 (PMC5639574; doi:10.1186/s13148-017-0415-6)
Supplement: Additional file 1: Table S1. — Clinicopathological characteristics of the study cohort. Table S2 Sequences and positions of oligonucleotides used in this study. Figure S1 KIBRA CpG islands were detected using “CpG Island searcher.” Two CpG islands were detected: CpG II with 205 bp and CpG I with 764 bp. Parameter settings: %GC = 55%, CpGobs/CpGexp = 0.65, lengths > 200 bp, distance = 100 bp. Position of KIBRA promoter regions P1b and P1a is indicated according to TSS1a (NM_015238). Figure S2 Hematoxylin and eosin staining from adjacent benign tissue (Ctrl; I/II) and ccRCC tissue (III/IV) used for the analysis (DOCX 3044 kb) [file 13148_2017_415_MOESM1_ESM.docx]

**Additional file 1**

**Table S1: TNM grading and characteristics of the study cohort.**

| **Features/ Variables** |  | **ccRCC** | **adjacent benign regions, control** |
| --- | --- | --- | --- |
| n |  | 32 | 32 |
| Age | years | 63 [40-87] | 66 [37-87] |
| Gender | female | 13 (40.6) | 14 (43.8) |
| Tumor stage | pT1 | 21 (65.6) | 11 (34.4) |
|  | pT2 | 3 (9.4) | 6 (18.8) |
|  | pT3 | 8 (25.0) | 10 (31.2) |
|  | n.a. | / | 5 (15.6) |
| Lymph nodes metastasis | N0 | 9 (28.1) | 8 (25.0) |
|  | N1 | / | 6 (18.8) |
|  | Nx | 12 (37.5) | 10 (31.2) |
|  | n.a. | 11 (34.4) | 8 (25.0) |
| Distant metastasis | M0 | 3 (9.4) | 4 (12.5) |
|  | M1 | 1 (3.1) | 2 (6.3) |
|  | Mx | 4 (12.5) | 13 (40.6) |
|  | n.a. | 24 (75.0) | 13 (40.6) |
| Tumor grade | G1 | 7 (21.9) | 3 (9.4) |
|  | G2 | 17 (53.1) | 19 (59.3) |
|  | G3 | 6 (18.8) | 4 (12.5) |
|  | n.a. | 2 (6.2) | 6 (18.8) |
| Invasion into lymph vessels | L0 | 28 (87.5) | 20 (62.5) |
|  | L1 | 1 (3.1) | 3 (9.4) |
|  | n.a. | 3 (9.4) | 9 (28.1) |
| Invasion into veins | V0 | 22 (68.8) | 14 (43.7) |
|  | V1 | 7 (21.9) | 11 (34.4) |
|  | n.a. | 3 (9.4) | 7 (21.9) |
| Resection status | R0 | 28 (87.5) | 20 (62.5) |
|  | R1 | 3 (9.4) | 4 (12.5) |
|  | n.a. | 1 (3.1) | 8 (25.0) |
| Status at 6 months follow-up | no tumor detected | 13 (40.6) | / |
|  | deceased^†^ | 1 (3.1) | / |
|  | n.a. | 18 (56.3) | 32 (100.0) |

Data are given as n (%) or mean (range). All tumor specimens were classified according to the UICC (Union Internationale Contre le Cancer) TNM staging system. n.a., not available; †, patient died of ccRCC.

**Table S2: Sequences and positions of oligonucleotides used in this study.**

| **Oligonucleotide** | **Sequence 5'- 3'** | **Position; ref. acc. #** |
| --- | --- | --- |
| **Oligonucleotide sequences used for promoter methylation analysis** | | |
| KIBRA_ss1 | TAATTTTATACGCGGGTAGTTAGAC | -3708; AC026689 |
| KIBRA_ss2 | GGTTTAGTTTTTTAGGTTTGGGA | -605; AC026689 |
| KIBRA_as1 | AAATTAAAACCTAAATCTATTCGCT | -3375; AC026689 |
| KIBRA_as2 | CAAATAAAAAACCCTAATAAAAACC | -127; AC026689 |
| T Easy_M13ss | CCCAGTCACGACGTTGTAAAACG | 2949-2972; pGEM-T Easy |
| T Easy_M13as | CCTGTGTGAAATTGTTATCCGCT | 176-197; pGEM-T Easy |
| **Oligonucleotide sequences used for real-time PCR** | | |
| KIBRA_ss3 | CTTCGACGGCAAGGTCTAC | exon 1/2; NM_015238 |
| KIBRA_as3 | GCGGTTTGGTGTACCTGTC | exon 1/2; NM_015238 |
| **Oligonucleotide sequences used for PCR** | | |
| KIBRA_ss6 | GACAGGTACACCAAACCGC | exon 1/2; NM_015238 |
| KIBRA_as6 | CTTCAGCTTGTTGACCCGG | exon 4/5; NM_015238 |

**Figure S1**


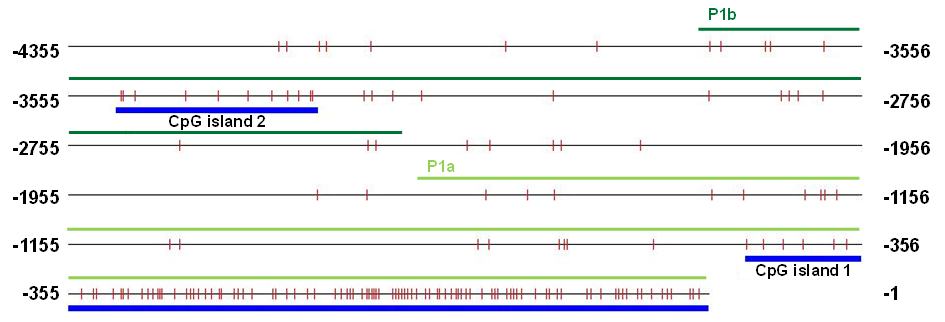


**Figure S1: *KIBR*A CpG islands were detected using ‘*CpG Island searcher’.*** Two CpG islands were detected: CpG II with 205 bp and CpG I with 764 bp. Parameter settings: %GC = 55%; CpG_obs_/CpG_exp_ = 0.65; lengths >200 bp; distance = 100 bp. Position of *KIBRA* promoter regions P1b and P1a is indicated according to TSS 1a (NM_015238).


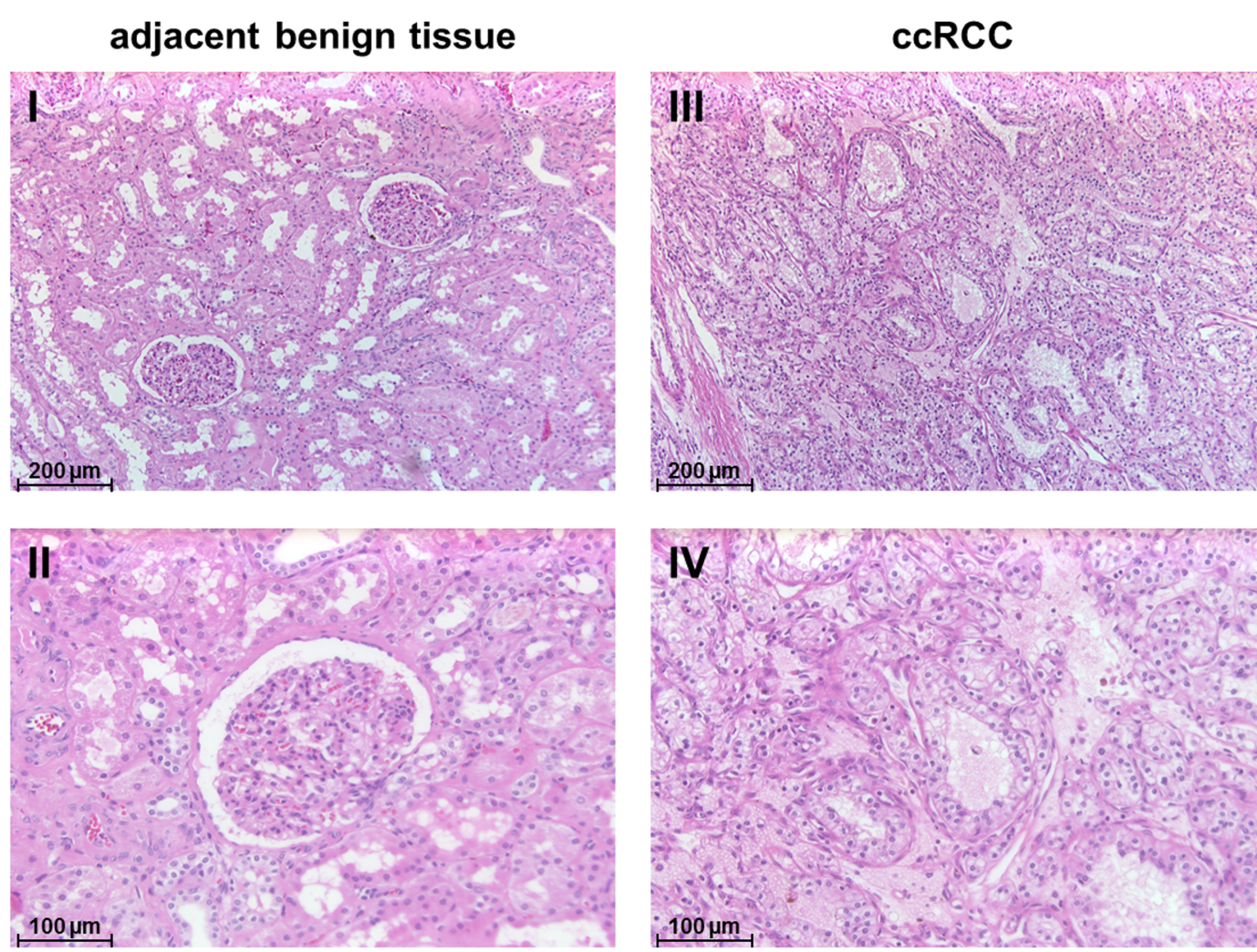


**Figure S2: Hematoxylin & eosin staining from adjacent benign tissue (Ctrl; I/II) and ccRCC tissue (III/IV) used for the analysis.**
